# Complement as a driver of immune–vascular heterogeneity across preeclampsia subtypes: toward a precision medicine framework

**DOI:** 10.3389/fimmu.2026.1855121

**Published:** 2026-06-26

**Authors:** Yafei Ge, Ting Gao

**Affiliations:** Department of Obstetrics and Gynecology, The Affiliated Hospital of Inner Mongolia Medical University, Hohhot, China

**Keywords:** biomarkers, complement system, endothelial dysfunction, inflammation, precision medicine, preeclampsia, pregnancy, subtype classification

## Abstract

Preeclampsia (PE) is a leading cause of maternal and perinatal morbidity worldwide, traditionally defined as a hypertensive disorder of pregnancy but increasingly recognized as a heterogeneous syndrome with diverse biological origins. Emerging evidence indicates that distinct pathogenic pathways—including placental insufficiency, maternal cardiometabolic dysfunction, and intrinsic immune dysregulation—contribute to different clinical phenotypes of the disease. Among these, the complement system has gained attention as a central regulator of immune–vascular interactions during pregnancy. Recent studies demonstrate that tightly controlled complement activation is required for normal placental development, whereas dysregulation of this system contributes to endothelial injury, inflammation, and microvascular dysfunction characteristic of PE. Importantly, complement activation patterns differ across disease subtypes: classical pathway activation predominates in placental-driven early-onset PE, chronic low-grade alternative pathway activation is associated with maternal metabolic disease, and genetic or functional defects in complement regulation define a subset of severe, complement-mediated cases with overlap features of thrombotic microangiopathy. Despite these advances, current diagnostic and therapeutic approaches remain largely non-specific and fail to account for this biological heterogeneity. Here, we propose a subtype-based framework of PE centered on complement dysregulation, integrating mechanistic, genetic, and clinical evidence. This model links distinct complement activation patterns to disease trajectories and identifies corresponding biomarker signatures and therapeutic targets. By redefining PE as a spectrum of complement-stratified disorders, this Review provides a conceptual foundation for precision diagnostics and mechanism-guided therapy. Such an approach has the potential to improve risk stratification, enable earlier detection, and support the development of targeted interventions tailored to individual disease mechanisms.

## 1 Introduction

Preeclampsia (PE) remains a major challenge in obstetric medicine, traditionally defined by new-onset hypertension and multi-organ dysfunction after 20 weeks of gestation. Increasing evidence indicates that this clinical definition encompasses biologically distinct disease entities rather than a single disorder. Early-onset PE is primarily driven by placental insufficiency, whereas late-onset PE is more closely associated with maternal cardiometabolic dysfunction, each exhibiting divergent vascular trajectories and long-term cardiovascular risks (1). These observations support a transition from phenotype-based classification toward mechanism-oriented stratification.

The complement system has emerged as a central regulator at the maternal–fetal interface. Rather than functioning solely as an innate immune effector cascade, it operates as a context-dependent immunomodulatory network that contributes to trophoblast invasion, vascular remodeling, and immune tolerance. Recent review articles have highlighted evidence supporting a role for spatially restricted complement activation in placental development and systemic complement dysregulation in endothelial injury (2). Complement activation fragments such as C3a and C5b-9 have been detected weeks before clinical onset, indicating a role in early disease initiation rather than secondary amplification (3).

**Figure 1:**
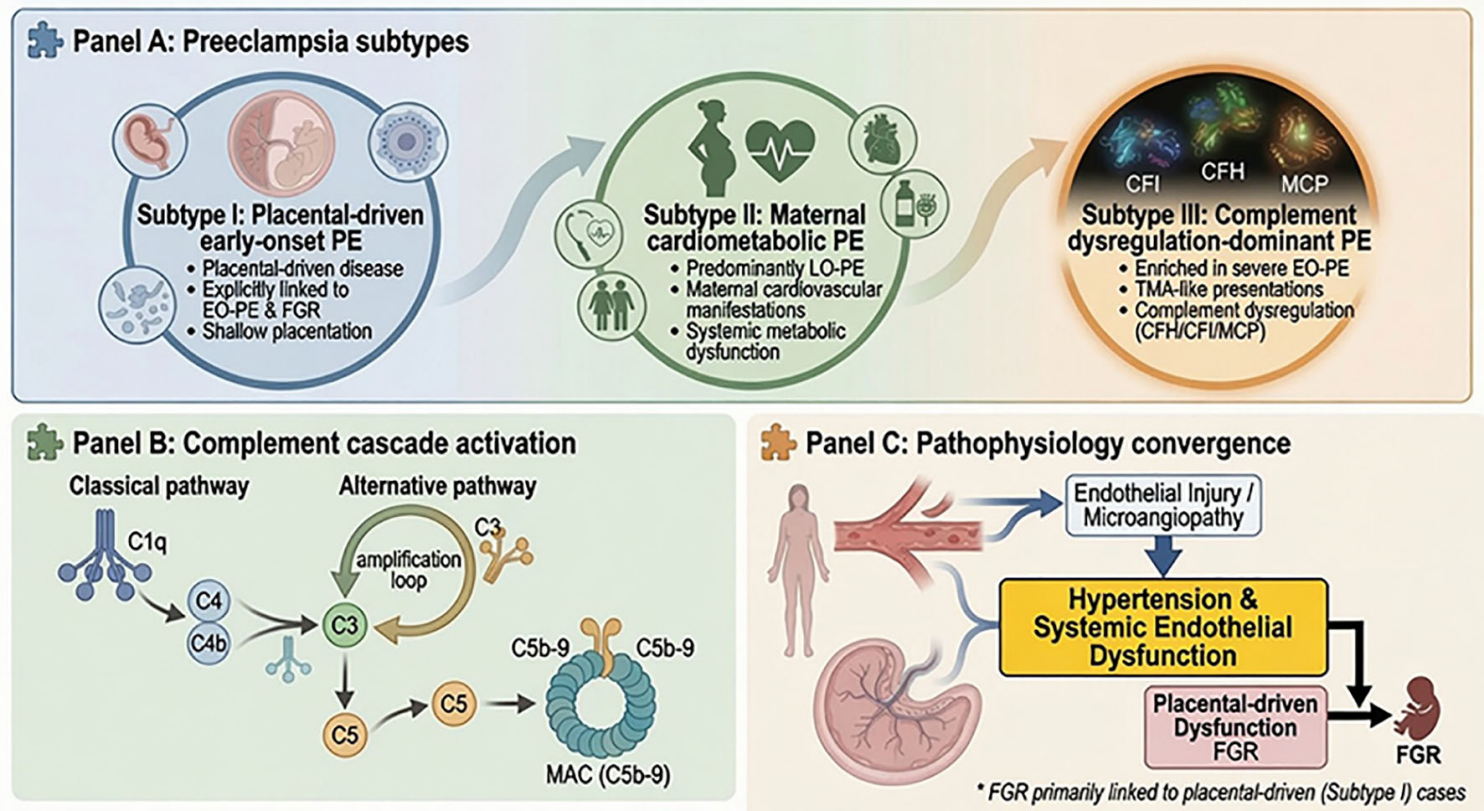
Integrated mechanistic framework of PE subtypes and complement-mediated pathogenesis. **(A)** Classification of PE mechanotypes. Preeclampsia is stratified into three distinct biological subtypes with clarified clinical phenotypes: (1) Subtype I (Placental-driven early-onset PE), explicitly linked to early-onset PE (EO-PE), shallow placentation, and fetal growth restriction (FGR); (2) Subtype II (Maternal cardiometabolic PE), predominantly linked to late-onset PE (LO-PE) and driven by maternal cardiovascular manifestations and systemic metabolic dysfunction; and (3) Subtype III (Complement dysregulation–dominant PE), arising from genetic susceptibility (e.g., CFH/CFI/MCP mutations) and enriched in severe early-onset and systemic thrombotic microangiopathy (TMA)-like presentations. **(B)** Complement cascade activation and amplification. Illustrates the convergent activation of the classical pathway (C1q/C4) and the alternative pathway amplification loop. These cascades culminate in the cleavage of C3 and C5, leading to the assembly of the terminal membrane attack complex (MAC/C5b-9), the primary effector of systemic cellular and endothelial injury. **(C)** Pathophysiological convergence and clinical outcomes. Depicts how diverse subtypic pathomechanisms and systemic complement activation converge downstream on endothelial injury, microangiopathy, and complement-mediated vascular damage. Hypertension and systemic endothelial dysfunction represent the principal downstream convergence points across subtypes. Notably, fetal growth restriction (FGR) is positioned as an important but subtype-dependent clinical outcome, driven primarily by placental dysfunction characteristic of Subtype I cases rather than serving as a universal manifestation of all PE subtypes.

Despite these advances, current models rarely integrate complement biology with clinical heterogeneity. Existing frameworks often fail to explain subtype-specific disease trajectories, account for mechanistic diversity, or guide targeted interventions (4). Although this review emphasizes complement biology, we acknowledge that PE pathogenesis involves complex interactions among multiple innate and adaptive immune pathways, including decidual NK-cell dysfunction, macrophage polarization, T-cell imbalance, B-cell–derived autoantibodies, inflammasome activation, and cytokine signaling networks. Complement activation may function either as a primary driver or as a downstream amplifier depending on the biological subtype and disease stage. Therefore, the framework proposed herein should be viewed as a complement-centered model of immune–vascular heterogeneity rather than a universal explanation for all PE cases. This Review proposes a subtype-based model in which PE is redefined as a complement-stratified syndrome, linking mechanistic pathways with clinical phenotypes to enable precision diagnosis and therapy. The conceptual framework of this Review is summarized in **Figure 1**.

## 2 Complement regulation in physiological pregnancy

### 2.1 Immune balance at the maternal–fetal interface

Successful pregnancy depends on a regulated immune equilibrium rather than immune suppression. At the maternal–fetal interface, trophoblasts interact with decidual immune cells within a microenvironment shaped by complement signaling. Review articles by Parker et al. and Wang et al. summarized evidence suggesting that controlled complement activation supports immune surveillance while limiting excessive inflammation (5, 6). Recent studies have further refined this paradigm. Agostinis et al. demonstrated that complement functions as a context-dependent immunomodulator rather than a purely effector cascade, integrating innate and adaptive immune responses at tissue interfaces (7). Crucially, complement signaling actively orchestrates the balance between pro-inflammatory and tolerogenic pathways, thereby enabling maternal immune adaptation (8).

Experimental animal studies provide important mechanistic evidence supporting the role of complement regulation in pregnancy. In C1q-deficient mice (9), impaired trophoblast invasion and defective spiral artery remodeling result in placental insufficiency, fetal growth restriction, and maternal hypertension-like features, highlighting the contribution of physiological C1q signaling to placental vascular development. Similarly, Crry-deficient mouse models provide direct evidence that complement regulation is essential at the maternal–fetal interface. Xu et al. demonstrated that loss of the murine complement regulator Crry leads to complement-mediated embryonic lethality, which can be rescued by concomitant C3 deficiency (10). More recently, placenta-specific Crry downregulation was shown to induce placental complement deposition, fetal growth restriction, and increased maternal blood pressure in mice, further supporting the relevance of complement regulatory failure to PE-like phenotypes (11). These findings provide direct evidence that tight complement regulation is essential for fetal survival and placental homeostasis. Together, these experimental models support a causal role for complement dysregulation in adverse pregnancy outcomes and provide mechanistic foundations for observations in human preeclampsia.

### 2.2 Controlled complement activation during placentation

Placentation requires low-level complement activation to support trophoblast invasion and vascular remodeling. Burwick et al. and Cheng et al. demonstrated that C3a and C5a promote angiogenesis and cellular migration (2, 12). Complement activity is tightly restricted spatially to prevent terminal pathway activation and tissue injury. Recent mechanistic studies have significantly deepened this understanding. Mechanistic studies have linked complement activation to angiogenic imbalance rather than simple pro-angiogenic stimulation. In a mouse model of immune-mediated pregnancy loss and fetal growth restriction, Girardi et al. demonstrated that complement activation, particularly C5a, induces dysregulation of angiogenic factors by increasing soluble VEGF receptor-1 and reducing available VEGF activity (13). Consistently, C5a-mediated trophoblast dysfunction has been associated with reduced PlGF and increased sFlt-1 expression in experimental studies of preeclampsia (14). Importantly, complement activation is spatially and temporally restricted, ensuring that terminal pathway activation remains tightly controlled and preventing excessive membrane attack complex (MAC)-mediated tissue injury under physiological conditions (15). These findings collectively redefine complement activation as an active regulator of placental development, and disruption of this finely tuned system provides a direct mechanistic bridge to PE pathogenesis.

### 2.3 Complement regulatory proteins in pregnancy

Complement homeostasis relies on regulatory proteins including CFH, CD55, and CD46. Recent studies have highlighted that impaired CFH-mediated regulation of the alternative pathway promotes excessive complement activation and endothelial injury, providing a mechanistic basis for complement dysregulation in pregnancy complications including preeclampsia (16). Recent genetic and functional investigations have further clarified the clinical relevance of these regulators. Józsi and Uzonyi demonstrated that partial CFH dysfunction amplifies alternative pathway activation under stress conditions (17). Similarly, Dessaix et al. reported that mutations in complement regulators predispose to pregnancy-associated thrombotic microangiopathy (18). Rodrigues et al. further showed that expression of these regulators is dynamically modulated during gestation, ensuring localized protection of trophoblast and endothelial cells (19). Collectively, impairment of these regulatory mechanisms represents a critical upstream event in complement dysregulation, forming the mechanistic basis for pathological activation in PE (**Table 1**).

**Table 1:** Complement regulation in normal pregnancy versus preeclampsia.

Component	Physiological role	PE change	Biological compartment	Gestational timing reported	Evidence type	Clinical consequence	Representative references
C1q	Facilitates trophoblast invasion, apoptotic cell clearance, and maternal–fetal immune tolerance	Placental C1q dysregulation (↓ expression and/or aberrant deposition)	Placenta, decidua	Mainly 2nd–3rd trimester	Human + Animal	Impaired spiral artery remodeling, placental inflammation	(7, 20)
C3 / C3a	Maintains controlled basal complement activation and angiogenesis	↑ C3a, alternative pathway amplification	Maternal plasma/serum	2nd–3rd trimester	Human	Endothelial dysfunction, systemic inflammation	(21)
C5a	Regulates immune signaling and vascular adaptation	↑ C5a	Maternal plasma/serum	Predominantly 3rd trimester	Human	Hypertension, leukocyte recruitment, vascular injury	(22, 23)
C5b-9 (MAC)	Restricted sublytic signaling involved in physiological tissue remodeling	↑ Circulating and placental deposition	Plasma, placenta, kidney	3rd trimester and severe PE	Human	Trophoblast injury, endothelial dysfunction, thrombotic microangiopathy	(22, 24)
CFH (Factor H)	Controls spontaneous alternative pathway activation	↓ Functional activity and/or pathogenic variants	Plasma, genetic analyses	Variable across gestation	Human	Uncontrolled complement activation, aHUS-like manifestations	(16, 17)
CD55 (DAF)	Accelerates decay of C3/C5 convertases	↓ Placental membrane expression	Placenta	Variable across gestation	Human	Amplified complement activation and inflammatory signaling	(16, 20)
MCP (CD46)	Cofactor for C3b/C4b degradation	↓ Regulatory capacity and pathogenic variants	Placenta, genetic analyses	Throughout pregnancy	Human	Local endothelial injury and alternative pathway dysregulation	(25, 26)

## 3 Subtype I: placental-driven early-onset preeclampsia

### 3.1 Placental hypoxia and trophoblast dysfunction

Early-onset preeclampsia (EO-PE) is a placenta-initiated disorder arising from defective trophoblast invasion and incomplete spiral artery remodeling, leading to chronic hypoxia and oxidative stress. These conditions impair trophoblast differentiation, increase apoptosis, and disrupt syncytialization, resulting in the release of anti-angiogenic and pro-inflammatory mediators. Human placental transcriptomic studies reported coordinated upregulation of hypoxia-inducible factor (HIF) signaling, complement activation genes, and innate immune pathways in EO-PE placentas (8, 27). Spatial transcriptomic analyses further refine this model. Agostinis et al. showed that hypoxic trophoblast regions exhibit localized enrichment of complement components, including C1q, C3, and factor B, indicating that complement activation is spatially organized rather than diffuse (7). Liu et al. reported that hypoxia shifts decidual macrophages toward a pro-inflammatory phenotype, reinforcing local complement activation (28). Together, these findings indicate that trophoblast dysfunction acts not only as a structural abnormality but also as an initiating immunological signal that promotes complement-mediated vascular injury.

### 3.2 Classical pathway activation as an upstream trigger

Placental injury in EO-PE promotes activation of the classical complement pathway. Increased trophoblast apoptosis releases damage-associated molecular patterns, including exposed phospholipids and nucleic acids, which facilitate C1q binding and initiate the complement cascade. Beyond its role in complement initiation, C1q contributes to trophoblast migration and vascular remodeling. Supporting evidence from C1q-deficient mouse models further demonstrated impaired trophoblast invasion, defective spiral artery remodeling, fetal growth restriction, and maternal hypertension-like features, highlighting the essential role of physiological C1q signaling in placental vascular development and pregnancy adaptation (9). However, excessive activation of the classical pathway drives downstream complement amplification and inflammatory signaling. Youssef et al. identified interactions between complement components and coagulation pathways, linking C1q with fibrinogen and platelet activation (29). These findings position the classical pathway as a key interface between placental injury and systemic vascular dysfunction.

### 3.3 Syncytiotrophoblast-derived complement amplification

The syncytiotrophoblast serves as the primary interface between maternal blood and fetal tissues and plays an active role in propagating complement activation. Under hypoxic stress, syncytiotrophoblasts release extracellular vesicles and apoptotic debris that stimulate complement activation in the maternal circulation. Govender et al. demonstrated that trophoblast-derived vesicles activate complement pathways (30), while Pierik et al. showed that reduced expression of complement regulatory proteins on trophoblast surfaces facilitates local amplification (31).

Recent reviews have summarized evidence suggesting that syncytiotrophoblast-derived extracellular vesicles activate the alternative complement pathway in maternal endothelial cells (32). Li et al. highlighted elevated circulating C3 and C5 activation fragments prior to clinical symptom onset, indicating early systemic involvement (33). These findings support the concept that the placenta functions as an active immunological signaling organ capable of initiating systemic complement activation.

### 3.4 Terminal pathway activation and endothelial injury

Terminal complement activation leads to formation of the membrane attack complex (C5b-9), a key mediator of endothelial and trophoblast injury. David et al. reported increased circulating and placental C5b-9 levels in preeclampsia, with strong associations with disease severity and organ dysfunction (22). Rather than inducing outright cell lysis, sublytic C5b-9 deposition promotes endothelial activation, oxidative stress, increased permeability, and a pro-thrombotic phenotype.

Recent studies have further clarified the pathogenic role of sublytic C5b-9 signaling in vascular dysfunction. Ramos et al. showed that C5b-9 deposition correlates with endothelial activation markers and predicts adverse maternal outcomes (24). Lu et al. highlighted evidence supporting that exposure to C5b-9 induces mitochondrial dysfunction and apoptotic signaling in trophoblasts under hypoxic conditions (34). Furthermore, Lin et al. reviewed evidence linking sublytic C5b-9 signaling to metabolic reprogramming in endothelial cells, highlighting a potential interaction between complement activation and cellular bioenergetics (35). Genetic studies summarized by Lokki et al. also support a contribution of terminal pathway dysregulation to hypertensive pregnancy disorders (36).

## 4 Subtype II: maternal cardiometabolic preeclampsia

### 4.1 Cardiometabolic dysfunction as the initiating driver

In contrast to placental-driven early-onset disease, Subtype II preeclampsia originates from pre-existing maternal cardiometabolic abnormalities, including obesity, chronic hypertension, insulin resistance, and dyslipidemia. Abdalla et al. and Chen et al. showed that these conditions establish a chronic low-grade inflammatory state characterized by altered adipokine signaling and persistent metabolic stress (37, 38). This systemic environment precedes pregnancy and primes the vasculature for exaggerated responses to gestational challenges.

Adipose tissue has emerged as an active immuno-metabolic organ in this context. Zhu et al. demonstrated sustained activation of C3 and C5 in obese adipose tissue independent of placental signals (39). Frimat et al. highlighted accumulating evidence that metabolic inflammation contributes to endothelial vulnerability to complement-mediated injury during pregnancy (40). In parallel, Jiménez-Osorio et al. reported that lipid peroxidation products and free fatty acids directly activate complement pathways, particularly the alternative pathway (41). Wojciuk et al. further demonstrated that adipose tissue produces complement components such as C3 and factor D, providing a systemic source of activation (42). These findings indicate that complement dysregulation can be established prior to placental involvement.

### 4.2 Persistent low-grade complement activation

Subtype II PE is characterized by sustained, low-level activation of the complement system rather than acute activation driven by placental injury. Copenhaver et al. demonstrated that elevated circulating C3 and C4 levels are closely associated with metabolic syndrome and chronic low-grade inflammation, supporting the concept that complement activation contributes to systemic innate immune activation in cardiometabolic disease (43). Metabolic stress further amplifies this process. Li et al. reviewed mechanistic evidence suggesting that insulin resistance may favor sustained C3 convertase activity and complement amplification (44). Balduit et al. identified complement activation patterns in metabolic PE that are distinct from angiogenic profiles associated with placental dysfunction (3). Prospective clinical cohort studies demonstrated that elevated C3 levels in early pregnancy predict subsequent development of PE (21). A systematic review by Ren et al. concluded that metabolic conditions enhance complement signaling through both increased activation and impaired regulation (45). Together, these findings support a model in which chronic metabolic stress sustains a self-perpetuating complement activation state.

### 4.3 Endothelial priming and heightened susceptibility

A defining feature of Subtype II PE is a pre-existing state of endothelial dysfunction. Chronic exposure to hyperglycemia, free fatty acids, and inflammatory cytokines leads to increased expression of adhesion molecules, impaired nitric oxide signaling, and a pro-thrombotic phenotype prior to pregnancy. Pluta et al. documented these vascular changes in individuals with cardiometabolic disease (46). Complement activation further enhances this vulnerability. Noris and Galbusera reviewed evidence indicating that C5a exposure promotes leukocyte adhesion and inflammatory gene expression in endothelial cells (47). Tang et al. reviewed evidence suggesting that sublytic complement activation induces epigenetic reprogramming of endothelial cells, increasing responsiveness to inflammatory stimuli (48). In addition, C5a signaling promotes endothelial adhesion molecule expression (VCAM-1, ICAM-1), facilitating leukocyte recruitment and vascular inflammation (49). Compared with Subtype I, where endothelial injury is largely a downstream consequence of placental-derived factors, Subtype II is characterized by pre-existing endothelial vulnerability, which amplifies the impact of pregnancy-related stressors. This distinction is critical for understanding disease heterogeneity and therapeutic targeting.

### 4.4 Integration with systemic inflammatory networks

Complement activation in Subtype II PE is closely integrated with systemic inflammatory signaling pathways. Xu et al. showed that C5a promotes cytokine release, leukocyte recruitment, and endothelial activation, contributing to amplification of inflammation (23). Recent multi-omics studies have clarified the molecular cross-talk between complement and inflammatory signaling pathways. Zhang et al. reviewed evidence suggesting that complement signaling synergizes with NF-κB activation to sustain chronic vascular inflammation (50). Mastellos et al. reviewed evidence supporting a bidirectional interaction between complement activation and inflammatory signaling pathways, whereby inflammatory mediators amplify complement activity while complement effector molecules further enhance cytokine production and leukocyte recruitment. (15) This interaction establishes a self-reinforcing inflammatory network linking metabolism, complement activation, and vascular dysfunction. In this model, maternal metabolic status serves as the primary determinant of disease trajectory, with placental involvement occurring as a secondary event.

## 5 Subtype III: complement dysregulation–dominant preeclampsia

### 5.1 Genetic defects in complement regulation (CFH, CFI, CD46)

A subset of preeclampsia represents a primary complement-mediated disorder in which intrinsic defects in complement regulation, rather than placental or metabolic triggers, dominate disease pathogenesis. Human genetic studies and translational investigations have shown that rare variants in complement regulatory genes—including *CFH*, *CFI*, and *CD46*—are enriched in patients with severe or atypical phenotypes (25). These genes encode key regulators of the alternative pathway, and loss-of-function variants impair control of spontaneous C3 activation, resulting in persistent complement amplification and heightened endothelial vulnerability.

Cohort studies by Livson et al. further demonstrate that a substantial proportion of patients with severe preeclampsia or thrombotic microangiopathy (TMA)-like features carry heterozygous variants in these regulatory pathways, supporting a genetically primed susceptibility state (26). This paradigm parallels atypical hemolytic uremic syndrome (aHUS), where complement dysregulation is a primary driver of endothelial injury (51).

Complement-mediated pregnancy complications are also supported by animal studies. Crry-deficient mice develop uncontrolled complement activation at the maternal–fetal interface and embryonic lethality, which can be rescued by concomitant C3 deficiency (10). These findings demonstrate the essential role of complement regulation in maintaining placental homeostasis.

Refined genomic analyses provide mechanistic insight into this susceptibility. Teoh et al. reported that CFH rare variants impair recognition of endothelial glycocalyx, leading to localized complement overactivation (52). Livson et al. showed that combined CFH–CFI variant burden correlates with earlier onset and increased disease severity in pregnancy-associated TMA (26). Together, these findings indicate that defective complement regulation actively determines endothelial sensitivity thresholds and supports the use of genetic screening for risk stratification in early-onset or refractory disease.

### 5.2 Alternative pathway overactivation loop

Uncontrolled activation of the alternative complement pathway is the central feature of this subtype. Under physiological conditions, low-level “tick-over” activation of C3 is tightly regulated. In the presence of CFH, CFI, or CD46 dysfunction, this process becomes dysregulated, leading to sustained C3 convertase activity and continuous downstream signaling (53). As a result, C3a and C5a are persistently generated, accompanied by accumulation of C5b-9 (membrane attack complex, MAC), which drives endothelial activation and injury independent of classical pathway triggers.

Experimental and translational studies support the role of the alternative pathway as a dominant amplification system. Harrison et al. highlighted evidence suggesting that alternative pathway activity is a major driver of terminal complement activation under dysregulated conditions (54). In parallel, Rawish et al. reviewed evidence linking complement activation to thromboinflammation and microvascular thrombosis through interactions with platelets, coagulation pathways, and endothelial injury (55). These observations position the alternative pathway as a central integrator of genetic susceptibility and environmental stress, and they provide a rationale for targeting upstream components such as C3 or factor D in selected patients.

### 5.3 Overlap with thrombotic microangiopathy

Complement dysregulation–dominant preeclampsia shows substantial overlap with TMA, which is characterized by microvascular thrombosis, hemolysis, thrombocytopenia, and organ injury (20). Palma et al. demonstrated that complement-mediated endothelial injury promotes platelet activation and fibrin deposition, establishing a mechanistic link between complement activation and TMA pathology (56).

This overlap complicates clinical differentiation between severe preeclampsia, HELLP syndrome, and complement-mediated TMA. Complement profiling provides additional discriminatory value. Clinical studies in pregnant women with TMA demonstrated that elevated soluble C5b-9 identifies complement-mediated disease (57). These findings suggest that a subset of clinically diagnosed preeclampsia represents misclassified complement-mediated TMA, with important therapeutic implications.

### 5.4 Continuum with atypical hemolytic uremic syndrome

Complement dysregulation–dominant preeclampsia lies on a disease continuum with aHUS, a prototypical complement-mediated TMA characterized by uncontrolled alternative pathway activation and systemic endothelial injury (58). Pregnancy is a recognized trigger for aHUS, and Gardikioti et al. showed that complement gene variants associated with aHUS are enriched in severe preeclampsia (59).

A unified conceptual framework has been proposed to link these conditions. Afshar-Kharghan suggested that complement-mediated pregnancy disorders represent a spectrum defined by the degree and timing of complement dysregulation (60). Within this model, disease phenotype ranges from preeclampsia-like presentations to overt aHUS, depending on activation thresholds. This continuum supports early identification of high-risk patients and consideration of complement-targeted intervention to prevent progression.

### 5.5 Severe phenotype clustering and clinical stratification

Clinically, Subtype III is associated with severe and atypical presentations, including early onset, rapid progression, and multiorgan involvement. Patients frequently exhibit TMA-like features such as thrombocytopenia, hemolysis, renal dysfunction, and refractory hypertension. Wong and Kavanagh reported that complement dysregulation defines a high-risk subgroup with poor clinical outcomes (61). Recent data-driven approaches have refined this classification. These findings support the concept that complement-mediated disease represents a distinct clinical entity rather than a continuum of severity. Stratification based on complement activation profiles may therefore improve risk prediction and guide targeted therapeutic selection.

## 6 Subtype-based biomarkers for precision diagnosis

### 6.1 Limitations of single-marker models

Current diagnostic strategies for preeclampsia primarily rely on angiogenic markers such as the sFlt-1/PlGF ratio, which reflect placental dysfunction but do not capture the biological heterogeneity of the disease (62, 63). This placenta-centric framework inherently assumes a dominant single-pathway mechanism and therefore performs best in early-onset, placental-driven disease, while showing reduced sensitivity in late-onset or metabolically driven phenotypes. Qi and Teschendorff reported substantial variability in biomarker performance across populations, indicating limited mechanistic specificity (64). In addition, angiogenic markers may remain within normal ranges in severe or atypical cases despite significant systemic disease activity.

Recent multi-cohort and systems biology studies have further reinforced these limitations by demonstrating that PE is not governed by a single dominant pathway but by interacting angiogenic, inflammatory, and complement-driven networks. Parapob et al. showed that angiogenic markers alone fail to identify up to one-third of clinically severe PE cases, particularly those without classical placental hypoxia signatures (65). These findings indicate that single-marker approaches do not adequately reflect upstream immune activation or downstream endothelial injury, contributing to delayed recognition of non-placental subtypes and missed opportunities for early intervention.

### 6.2 Subtype-specific biomarker signatures

Accumulating evidence supports the existence of distinct biomarker profiles aligned with mechanistically defined preeclampsia subtypes. In placental-driven disease (Subtype I), angiogenic imbalance remains the dominant feature. Domínguez del Olmo et al. showed that elevated sFlt-1 and reduced PlGF reflect impaired trophoblast function and placental hypoxia, providing a reliable signature for early-onset disease (62).

In contrast, maternal cardiometabolic preeclampsia (Subtype II) is characterized by systemic inflammatory activation. David et al. reported that complement fragments such as C3a and C5a, together with pro-inflammatory cytokines including IL-6 and TNF-α, are elevated even in the absence of overt placental pathology, consistent with chronic immune priming driven by metabolic dysfunction (22).

Complement dysregulation–dominant disease (Subtype III) exhibits a distinct profile centered on failure of alternative pathway regulation and terminal complement activation. Burwick et al. and Musalem et al. demonstrated that reduced CFH activity, variants in CFI and CD46, and elevated C5b-9 levels are strongly associated with severe phenotypes and thrombotic microangiopathy overlap (16, 66). These observations support a direct pathogenic role of complement activation rather than a secondary response.

High-dimensional proteomic and multi-omics studies further reinforce this framework by showing that each subtype is characterized by dominance of a specific biological axis—angiogenic, inflammatory, or complement-mediated—thereby enabling a shift from phenotype-based diagnosis toward mechanism-based classification.

### 6.3 Complement-based diagnostic stratification

The complement system provides an integrative biomarker axis that links innate immune activation, endothelial injury, and angiogenic imbalance across preeclampsia subtypes. Unlike conventional markers that reflect isolated processes, complement components capture both early activation events and downstream tissue injury, making them particularly suitable for subtype-level stratification.

Translational studies have demonstrated the added value of complement markers in diagnostic models. Burwick et al. highlighted evidence indicating that circulating complement activation products, including C3a, C5a, and soluble C5b-9, are associated with disease severity and may provide mechanistic information beyond conventional angiogenic biomarkers (16). Similarly, Balduit et al. systematically reviewed the available evidence and concluded that complement components show promise as biomarkers for identifying biologically distinct preeclampsia phenotypes, particularly when integrated with established angiogenic markers and clinical risk factors (3). These findings suggest that complement profiling may improve subtype classification by capturing immune activation, endothelial injury, and complement dysregulation that are not fully reflected by angiogenic markers alone.

These findings support a stratification framework based on three complementary biological layers: angiogenic imbalance reflecting placental dysfunction, systemic inflammation captured by C3a and C5a, and terminal complement activation indicated by C5b-9. Integrating these dimensions provides a foundation for precision diagnostics, enabling earlier detection, improved risk stratification, and more targeted therapeutic decision-making (**Table 2**).

**Table 2:** Biomarker signatures across preeclampsia subtypes and their clinical utility.

Biomarker	Subtype association	Change in PE	Biological compartment	Gestational timing	Pathophysiological meaning	Clinical utility	Representative references
sFlt-1/PlGF ratio	Subtype I	↑ sFlt-1, ↓ PlGF	Maternal plasma/serum	Mainly 2nd–3rd trimester	Angiogenic imbalance and placental hypoxia	Differential diagnosis and short-term prediction of delivery	(1, 62)
Soluble Endoglin (sEng)	Subtype I	↑	Maternal plasma/serum	2nd–3rd trimester	Severe endothelial dysfunction and impaired TGF-β signaling	Assessment of disease severity and multi-organ involvement	(63)
C5b-9 (sMAC)	Subtype III > Subtype I	↑↑	Plasma/serum	Predominantly 3rd trimester	Terminal complement activation and microvascular injury	Indicator of severe disease and adverse maternal–fetal outcomes	(22, 24)
C3a	Subtype II	↑	Maternal plasma/serum	Early to late pregnancy	Chronic inflammatory priming and alternative pathway activation	Early risk stratification and metabolic PE identification	(21)
C5a	Subtype II	↑	Maternal plasma/serum	Mid-to-late pregnancy	Systemic inflammatory amplification and endothelial activation	Monitoring inflammatory disease activity	(15, 23)
CFH Activity / CFH Variants	Subtype III	↓ activity and/or pathogenic variants	Plasma and genomic testing	Throughout pregnancy	Failure of alternative pathway regulation	Genetic risk assessment and identification of complement-mediated PE	(16)
CFI / MCP (CD46) Variants	Subtype III	Pathogenic variants enriched	Genomic testing	Throughout pregnancy	Impaired control of complement amplification loop	Precision subtype classification and therapeutic selection	(25, 26)
IL-6 / TNF-α	Subtype II	↑	Plasma/serum	Early to late pregnancy	Low-grade systemic inflammation and metabolic immune activation	Adjunct prediction of cardiometabolic complications	(37, 38)

## 7 Subtype-based therapeutic strategies and precision medicine

### 7.1 Limitations of uniform treatment strategies

Current management of preeclampsia remains largely non-specific, focusing on blood pressure control, seizure prophylaxis, and delivery as the only definitive intervention (1, 67). Although these approaches reduce acute maternal risk, they do not address the underlying biological mechanisms, which differ substantially across disease subtypes. This uniform strategy is based on the implicit assumption that preeclampsia represents a single disease entity, resulting in variable treatment responses and limited efficacy, particularly in early-onset and severe cases.

Clinical and systems-level studies highlight the limitations of this approach. Story et al. reported that patients with complement-driven or TMA-like disease show reduced responsiveness to conventional antihypertensive management and delivery-based strategies (68). Burwick and Rodriguez further demonstrated that prevention models based solely on angiogenic markers do not reduce adverse outcomes in metabolically driven populations (2). These observations indicate that current management does not target the dominant pathogenic axis—placental, metabolic, or complement-mediated—leaving a substantial therapeutic gap, especially in Subtype II and III disease.

### 7.2 Complement-targeted therapies

Complement inhibition has emerged as a mechanism-based therapeutic strategy, particularly in complement dysregulation–dominant and TMA-like disease. The terminal component C5 represents a key convergence point in the complement cascade, and its blockade prevents generation of both C5a and C5b-9, thereby limiting endothelial injury and inflammatory amplification. Fakhouri et al. and Smith-Jackson and Harrison demonstrated the efficacy of the anti-C5 antibody eculizumab in complement-mediated disorders, including aHUS and pregnancy-associated TMA (69, 70).

Subsequent studies extend these findings to preeclampsia. Sperati reported sustained renal and hematologic improvement in complement-mediated TMA treated with C5 inhibition (71), while Frimat et al. described successful pregnancy prolongation in selected severe cases with documented complement overactivation (40). New therapeutic agents are also being developed. Long-acting C5 inhibitors such as ravulizumab and upstream inhibitors targeting C3 or factor D aim to achieve broader control of complement amplification (72). These approaches suggest that treatment efficacy is closely linked to underlying disease biology, with the greatest benefit observed in patients with genetic complement dysregulation or TMA-like features.

### 7.3 Anticoagulation and endothelial stabilization

Given the central role of endothelial dysfunction and microvascular injury, therapies targeting vascular integrity and coagulation pathways represent an additional treatment axis. Low-molecular-weight heparin (LMWH) has both anticoagulant and anti-inflammatory properties and has been shown to partially inhibit complement activation while preserving endothelial glycocalyx structure (73). Statins, particularly pravastatin, have been investigated for their ability to restore angiogenic balance, reduce oxidative stress, and improve endothelial function (74, 75).

Experimental studies provide mechanistic support for these effects. Kolev et al. highlighted the central role of C3 activation as a therapeutic target for controlling complement amplification and downstream inflammatory injury, underscoring the importance of interventions capable of modulating complement activity in thromboinflammatory diseases (76), while Iannaccone et al. showed that pravastatin reduces circulating sFlt-1 levels and improves endothelial stability in high-risk pregnancies (77). These interventions primarily modulate upstream vascular and inflammatory processes and may therefore be more effective in placental-driven and metabolic subtypes, whereas their impact appears limited in complement-driven disease.

Low-dose aspirin remains the cornerstone of primary prevention for preeclampsia in women at increased risk and is recommended by major international guidelines (1). Although its established mechanism involves inhibition of platelet cyclooxygenase-1 activity and suppression of thromboxane A_2_ production, emerging evidence suggests that platelet-mediated thromboinflammatory pathways may intersect with complement activation. Experimental studies have demonstrated that activated platelets can directly trigger complement activation and amplify inflammatory responses (78). In addition, complement activation interacts extensively with platelet function, coagulation pathways, and endothelial injury, forming a tightly integrated thromboinflammatory network. Therefore, aspirin may indirectly attenuate complement–coagulation cross-talk by reducing platelet activation and downstream vascular inflammation. However, direct clinical evidence demonstrating meaningful modulation of complement activity by aspirin in preeclampsia remains limited. Future studies should determine whether complement biomarkers can identify patient subsets that derive differential benefit from aspirin prophylaxis and whether combined targeting of complement and platelet pathways may further improve preventive strategies.

### 7.4 Plasma exchange in severe disease

Therapeutic plasma exchange (TPE) is used as a rescue strategy in severe or refractory cases, particularly when features of thrombotic microangiopathy are present. This approach removes circulating complement components, autoantibodies, and inflammatory mediators, while restoring functional regulatory proteins such as CFH (79). Demel et al. reported rapid improvement in hematologic and renal parameters in selected patients with severe disease (80).

Recent studies have refined its clinical application. Azoulay et al. highlighted clinical reports supporting the combined use of therapeutic plasma exchange and complement inhibition in refractory complement-mediated TMA (81). Despite these benefits, TPE lacks pathway specificity and is generally reserved for critically ill patients or as a bridging therapy when targeted treatments are unavailable.

### 7.5 Toward precision obstetric immunotherapy

Advances in subtype classification, biomarker profiling, and targeted therapies are reshaping preeclampsia management toward a precision medicine framework. Future strategies are likely to integrate angiogenic modulation, anti-inflammatory approaches, and complement inhibition according to individual disease mechanisms. Technologies such as multi-omics profiling and machine learning are enabling earlier identification of high-risk patients and dynamic stratification across gestation (82).

Conceptual and translational studies support this transition. Bachnas et al. highlighted the feasibility of multi-omics–guided precision medicine in pregnancy disorders (83). Kumar and Stewart identified emerging inhibitors targeting C3 and factor D as next-generation therapeutics capable of modulating upstream complement activation (32). These developments enable intervention at multiple levels of disease biology, from placental dysfunction to systemic inflammation and terminal vascular injury, and mark a shift from reactive management to mechanism-based prevention and treatment.

## 8 Conclusion and future perspectives

PE is increasingly recognized as a heterogeneous syndrome comprising biologically distinct subtypes rather than a single disease entity. In this review, we propose a mechanistic framework in which PE is stratified into placental-driven, maternal cardiometabolic, and complement dysregulation–dominant subtypes, each characterized by unique patterns of immune–vascular interaction.

Although multiple innate and adaptive immune pathways contribute to PE pathogenesis, accumulating evidence suggests that complement activation represents an important integrative mechanism linking placental dysfunction, systemic inflammation, endothelial injury, and thrombo-inflammatory responses. Within this context, complement activity may function either as a primary pathogenic driver or as a downstream amplifier depending on the biological subtype and disease stage. Accordingly, the proposed framework should be viewed as a complement-centered model of immune–vascular heterogeneity rather than a universal explanation for all PE cases.

Despite these conceptual advances, translation into clinical practice remains limited. Most current evidence is derived from retrospective or cross-sectional studies, which constrain the ability to define causal relationships and longitudinal disease trajectories. Prospective, multi-omics cohort studies integrating complement activation profiles, genomic susceptibility, and longitudinal clinical data are therefore required. Such approaches will be essential to validate subtype classification, establish robust biomarker thresholds, and develop clinically actionable risk models. Future investigations should also clarify how complement signaling interacts with other key immune pathways, including decidual NK-cell biology, macrophage polarization, T-cell regulation, B-cell–mediated autoimmunity, and inflammasome activation. Understanding these interactions will be critical for defining the relative contribution of complement-dependent and complement-independent mechanisms across PE subtypes. In particular, standardized assessment of complement activity across gestation will be critical for linking mechanistic insights with real-time clinical decision-making.

Future progress will depend on the transition toward precision obstetric immunology, in which diagnosis and treatment are guided by underlying biological mechanisms rather than syndromic definitions. Advances in complement-targeted therapies, biomarker-based stratification, and computational modeling are beginning to enable this shift. However, complement-directed approaches should be integrated within broader immune and vascular frameworks that acknowledge the multifactorial nature of PE. Such integration may facilitate earlier identification of high-risk patients, improve therapeutic precision, and enable individualized management strategies. Beyond improving maternal and fetal outcomes in PE, this framework may also provide a broader model for precision medicine in pregnancy-associated inflammatory disorders.

## References

[1] Chappell LC Cluver CA Kingdom J Tong S . Pre-eclampsia. Lancet. (2021) 398:341–54. doi: 10.1016/s0140-6736(20)32335-7 34051884

[2] Burwick RM Rodriguez MH . Angiogenic biomarkers in preeclampsia. Obstet Gynecol. (2024) 143:515–23. doi: 10.1097/aog.0000000000005532 38350106

[3] Balduit A Agostinis C Mangogna A Zito G Stampalija T Ricci G . Systematic review of the complement components as potential biomarkers of pre-eclampsia: pitfalls and opportunities. Front Immunol. (2024) 15:1419540. doi: 10.3389/fimmu.2024.1419540 38983853 PMC11232388

[4] Miller D Motomura K Galaz J Gershater M Lee ED Romero R . Cellular immune responses in the pathophysiology of preeclampsia. J Leukoc Biol. (2022) 111:237–60. doi: 10.1002/jlb.5ru1120-787rr 33847419 PMC8511357

[5] Parker EL Silverstein RB Verma S Mysorekar IU . Viral-immune cell interactions at the maternal-fetal interface in human pregnancy. Front Immunol. (2020) 11:522047. doi: 10.3389/fimmu.2020.522047 33117336 PMC7576479

[6] Wang J Han T Zhu X . Role of maternal-fetal immune tolerance in the establishment and maintenance of pregnancy. Chin Med J (Engl). (2024) 137:1399–406. doi: 10.1097/cm9.0000000000003114 38724467 PMC11188918

[7] Agostinis C Mangogna A Balduit A Kishore U Bulla R . A non-redundant role of complement protein C1q in normal and adverse pregnancy. Explor Immunol. (2022) 2:622–36. doi: 10.37349/ei.2022.00072

[8] Alippe Y Hatterschide J Coyne CB Diamond MS . Innate immune responses to pathogens at the maternal-fetal interface. Nat Rev Immunol. (2025) 25:869–84. doi: 10.1038/s41577-025-01191-0 40533582

[9] Agostinis C Bulla R Tripodo C Gismondi A Stabile H Bossi F . An alternative role of C1q in cell migration and tissue remodeling: contribution to trophoblast invasion and placental development. J Immunol. (2010) 185:4420–9. doi: 10.4049/jimmunol.0903215 20810993

[10] Xu C Mao D Holers VM Palanca B Cheng AM Molina H . A critical role for murine complement regulator crry in fetomaternal tolerance. Science. (2000) 287:498–501. doi: 10.1126/science.287.5452.498 10642554

[11] Banadakoppa M Pennington K Balakrishnan M Yallampalli C . Complement inhibitor Crry expression in mouse placenta is essential for maintaining normal blood pressure and fetal growth. PloS One. (2020) 15:e0236968. doi: 10.1371/journal.pone.0236968 32745140 PMC7398533

[12] Cheng S Norris W Kalkunte S Jash S Richardson LR Sharma S . Evidence for complement activation in preeclampsia placenta and its presence in circulation. Am J Reprod Immunol. (2025) 93:e70076. doi: 10.1111/aji.70076 40388260

[13] Girardi G Yarilin D Thurman JM Holers VM Salmon JE . Complement activation induces dysregulation of angiogenic factors and causes fetal rejection and growth restriction. J Exp Med. (2006) 203:2165–75. doi: 10.1084/jem.20061022 16923853 PMC2118387

[14] Ma Y Kong LR Ge Q Lu YY Hong MN Zhang Y . Complement 5a-mediated trophoblasts dysfunction is involved in the development of pre-eclampsia. J Cell Mol Med. (2018) 22:1034–46. doi: 10.1111/jcmm.13466 29168351 PMC5783881

[15] Mastellos DC Hajishengallis G Lambris JD . A guide to complement biology, pathology and therapeutic opportunity. Nat Rev Immunol. (2024) 24:118–41. doi: 10.1038/s41577-023-00926-1 37670180

[16] Burwick RM Java A Regal JF . The role of complement in normal pregnancy and preeclampsia. Front Immunol. (2025) 16:1643896. doi: 10.3389/fimmu.2025.1643896 40777009 PMC12328418

[17] Józsi M Uzonyi B . Factor H-related proteins and their associated disorders. J Allergy Clin Immunol. (2025) 156:311–4. doi: 10.1016/j.jaci.2025.05.024 40499620

[18] Dessaix K Bontoux C Aubert O Grünenwald A Sberro Soussan R Zuber J . De novo thrombotic microangiopathy after kidney transplantation in adults: interplay between complement genetics and multiple endothelial injury. Am J Transplant. (2024) 24:1205–17. doi: 10.1016/j.ajt.2024.01.029 38320731

[19] Rodrigues SG Assis KA Silva JLM Privado DJT Alves JV Faulkner JL . Molecular signatures of preeclampsia: the role of post-translational protein modifications. Compr Physiol. (2026) 16:e70107. doi: 10.1002/cph4.70107 41622532

[20] Lokki AI Heikkinen-Eloranta J . Pregnancy induced TMA in severe preeclampsia results from complement-mediated thromboinflammation. Hum Immunol. (2021) 82:371–8. doi: 10.1016/j.humimm.2021.03.006 33820656

[21] Starodubtseva N Tokareva A Kononikhin A Brzhozovskiy A Bugrova A Kukaev E . First-trimester preeclampsia-induced disturbance in maternal blood serum proteome: a pilot study. Int J Mol Sci. (2024) 25:10653. doi: 10.3390/ijms251910653 39408980 PMC11476624

[22] David M Maharaj N Krishnan A . Exosomal-complement system activation in preeclampsia. J Obstet Gynaecol Res. (2025) 51:e16255. doi: 10.1111/jog.16255 40070019 PMC11897585

[23] Xu B Zhou Z Xiao Y Liu Q Xiao T Lv Z . The pleiotropic effect of complement C5a-C5aR1 pathway in diseases: from immune regulation to targeted therapy. Int J Mol Sci. (2025) 26:11693. doi: 10.3390/ijms262311693 41373839 PMC12691729

[24] Ramos A Youssef L Molina P Martinez-Sanchez J Moreno-Castaño AB Blasco M . Endothelial damage and complement dysregulation in fetuses from pregnancies complicated by preeclampsia. Acta Obstet Gynecol Scand. (2025) 104:829–38. doi: 10.1111/aogs.15072 40007223 PMC11981108

[25] Salmon JE Heuser C Triebwasser M Liszewski MK Kavanagh D Roumenina L . Mutations in complement regulatory proteins predispose to preeclampsia: a genetic analysis of the PROMISSE cohort. PloS Med. (2011) 8:e1001013. doi: 10.1371/journal.pmed.1001013 21445332 PMC3062534

[26] Livson S Heikkinen-Eloranta J Messing M Lokki AI Meri S . Pregnancy-related thrombotic microangiopathy has a spectrum of underlying causes. Pregnancy Hypertens. (2025) 40:101212. doi: 10.1016/j.preghy.2025.101212 40306205

[27] Stoiber M Horvat Mercnik M Hirschmugl B Darnhofer B Pernitsch D Leopold-Posch B . Proteomic epithelial-to-mesenchymal transition signature in fetoplacental small extracellular vesicles of early-onset preeclampsia. J Extracell Biol. (2026) 5:e70122. doi: 10.1002/jex2.70122 41788817 PMC12957779

[28] Liu L Li X Yang H Xu F Dong X . Bioinformatic analysis of apoptosis-related genes in preeclampsia using public transcriptomic and single-cell RNA sequencing datasets. J Inflammation Res. (2025) 18:4785–812. doi: 10.2147/jir.S507660 40224388 PMC11992479

[29] Youssef L Miranda J Blasco M Paules C Crovetto F Palomo M . Complement and coagulation cascades activation is the main pathophysiological pathway in early-onset severe preeclampsia revealed by maternal proteomics. Sci Rep. (2021) 11:3048. doi: 10.1038/s41598-021-82733-z 33542402 PMC7862439

[30] Govender S David M Naicker T . Is the complement system dysregulated in preeclampsia comorbid with HIV infection? Int J Mol Sci. (2024) 25:6232. doi: 10.3390/ijms25116232 38892429 PMC11172754

[31] Pierik E de Jong-Schoots MH Bulthuis M Daha MR van den Born J Gordijn SJ . Preeclampsia is associated with placental complement C4d deposition. Placenta. (2025) 171:45–51. doi: 10.1016/j.placenta.2025.09.001 40966963

[32] Kumar V Stewart JHT . The complement system in human pregnancy and preeclampsia. Front Immunol. (2025) 16:1617140. doi: 10.3389/fimmu.2025.1617140 40904461 PMC12401976

[33] Li Q Wei X Zhang Y Zeng W Lin Y . Trophoblast adaptation to hypoxia: balance and dysfunction. Cell Commun Signal. (2025) 23:538. doi: 10.1186/s12964-025-02531-2 41462473 PMC12751209

[34] Lu L Huang X Shi Y Jiang Y Han Y Zhang Y . Mitochondrial dysfunction in pregnancy loss: a review. Mol Cell Biochem. (2025) 480:2749–64. doi: 10.1007/s11010-024-05171-1 39621222

[35] Lin JC Hwang SW Luo H Mohamud Y . Double-edged sword: exploring the mitochondria-complement bidirectional connection in cellular response and disease. Biol (Basel). (2024) 13:431. doi: 10.3390/biology13060431 38927311 PMC11200454

[36] Lokki AI Triebwasser M Daly E Kurki MI Perola M Auro K . Understanding rare genetic variants within the terminal pathway of complement system in preeclampsia. Genes Immun. (2025) 26:22–6. doi: 10.1038/s41435-024-00310-6 39690307 PMC11832413

[37] Abdalla RAH Parveen N Iqbal N Mohamed AAA Shahid SMA Elhussein G . Adipokines in preeclampsia: disrupted signaling pathways and novel therapeutic strategies. Eur J Med Res. (2025) 30:702. doi: 10.1186/s40001-025-02972-y 40759967 PMC12320359

[38] Chen Y Wu L Huang D Wu X Liu K Sun B . Subtype specific immune-metabolic reprogramming in preeclampsia revealed by multiomics and serum biomarkers. Hypertens Res. (2026) 49:641–57. doi: 10.1038/s41440-025-02504-5 41419624 PMC12960252

[39] Zhu S Chen S Ge Y Zhou F Su K Xu C . High-fat diet induces pre-eclampsia through dampening cell-autonomous C3 in trophoblasts. Commun Biol. (2025) 8:879. doi: 10.1038/s42003-025-08298-z 40481230 PMC12144106

[40] Frimat M Gnemmi V Stichelbout M Provôt F Fakhouri F . Pregnancy as a susceptible state for thrombotic microangiopathies. Front Med (Lausanne). (2024) 11:1343060. doi: 10.3389/fmed.2024.1343060 38476448 PMC10927739

[41] Jiménez-Osorio AS Carreón-Torres E Correa-Solís E Ángel-García J Arias-Rico J Jiménez-Garza O . Inflammation and oxidative stress induced by obesity, gestational diabetes, and preeclampsia in pregnancy: role of high-density lipoproteins as vectors for bioactive compounds. Antioxidants (Basel). (2023) 12:1894. doi: 10.3390/antiox12101894 37891973 PMC10604737

[42] Wojciuk B Frulenko I Brodkiewicz A Kita D Baluta M Jędrzejczyk F . The complement system as a part of immunometabolic post-exercise response in adipose and muscle tissue. Int J Mol Sci. (2024) 25:11608. doi: 10.3390/ijms252111608 39519159 PMC11545998

[43] Copenhaver M Yu CY Hoffman RP . Complement components, C3 and C4, and the metabolic syndrome. Curr Diabetes Rev. (2019) 15:44–8. doi: 10.2174/1573399814666180417122030 29663892

[44] Li Y Zeng H Zhong X . Complement C3 in panvascular disease: a central integrator of immune signaling and vascular remodeling. Clin Sci (Lond). (2025) 139:1373–403. doi: 10.1042/cs20257865 41187099 PMC12687451

[45] Ren J Yang M Zhu Q Wang M Li N . Pathophysiologic mechanisms and molecular targets in preeclampsia. Clin Chim Acta. (2026) 586:120885. doi: 10.1016/j.cca.2026.120885 41698634

[46] Pluta W Lubkowska A Dudzińska W . Vascular endothelium in health and disease: structure, function, assessment and role in metabolic disorders. Vasc Health Risk Manag. (2025) 21:729–47. doi: 10.2147/vhrm.S519426 40927226 PMC12415623

[47] Noris M Galbusera M . The complement alternative pathway and hemostasis. Immunol Rev. (2023) 313:139–61. doi: 10.1111/imr.13150 36271870

[48] Tang L Li L Liu Y Zhang X Wang X Zheng Z . Epigenetic modifications in vascular inflammation. Front Immunol. (2025) 16:1711579. doi: 10.3389/fimmu.2025.1711579 41425580 PMC12711845

[49] Yan Y Wang L Zhong N Wen D Liu L . Multifaced roles of adipokines in endothelial cell function. Front Endocrinol (Lausanne). (2024) 15:1490143. doi: 10.3389/fendo.2024.1490143 39558976 PMC11570283

[50] Zhang K Sang J Kan C Sheng S Sun X Guo Z . STAT signaling in metabolic disorders: from molecular mechanisms to therapeutic implications. Cell Biochem Biophys. (2026) 84:1861–74. doi: 10.1007/s12013-026-02038-8 41739314

[51] Avdonin PP Blinova MS Generalova GA Emirova KM Avdonin PV . The role of the complement system in the pathogenesis of infectious forms of hemolytic uremic syndrome. Biomolecules. (2023) 14:39. doi: 10.3390/biom14010039 38254639 PMC10813406

[52] Teoh CW Riedl Khursigara M Ortiz-Sandoval CG Park JW Li J Bohorquez-Hernandez A . The loss of glycocalyx integrity impairs complement factor H binding and contributes to cyclosporine-induced endothelial cell injury. Front Med (Lausanne). (2023) 10:891513. doi: 10.3389/fmed.2023.891513 36860338 PMC9968885

[53] Li J Wang K Starodubtseva MN Nadyrov E Kapron CM Hoh J . Complement factor H in molecular regulation of angiogenesis. Med Rev (2021). (2024) 4:452–66. doi: 10.1515/mr-2023-0048 39444793 PMC11495524

[54] Harrison RA Harris CL Thurman JM . The complement alternative pathway in health and disease-activation or amplification? Immunol Rev. (2023) 313:6–14. doi: 10.1111/imr.13172 36424888

[55] Rawish E Sauter M Sauter R Nording H Langer HF . Complement, inflammation and thrombosis. Br J Pharmacol. (2021) 178:2892–904. doi: 10.1111/bph.15476 33817781

[56] Palma LMP Sridharan M Sethi S . Complement in secondary thrombotic microangiopathy. Kidney Int Rep. (2021) 6:11–23. doi: 10.1016/j.ekir.2020.10.009 33102952 PMC7575444

[57] Liu X Hu Y Yu X Tan Y Yu F Chen M . Differential contributions of the C5b-9 and C5a/C5aR pathways to microvascular and macrovascular thrombosis in complement-mediated thrombotic microangiopathy patients. Clin Immunol. (2024) 259:109871. doi: 10.1016/j.clim.2023.109871 38101498

[58] Bogdan RG Anderco P Ichim C Cimpean AM Todor SB Glaja-Iliescu M . Atypical hemolytic uremic syndrome: a review of complement dysregulation, genetic susceptibility and multiorgan involvement. J Clin Med. (2025) 14:2527. doi: 10.3390/jcm14072527 40217974 PMC11989465

[59] Gardikioti A Venou TM Gavriilaki E Vetsiou E Mavrikou I Dinas K . Molecular advances in preeclampsia and HELLP syndrome. Int J Mol Sci. (2022) 23:3851. doi: 10.3390/ijms23073851 35409211 PMC8999044

[60] Afshar-Kharghan V . Long-term outcome and management of complement-mediated thrombotic microangiopathy/aHUS. Hematol Am Soc Hematol Educ Program. (2025) 2025:147–53. doi: 10.1182/hematology.2025000700C 41347985 PMC12897940

[61] Wong EKS Kavanagh D . Diseases of complement dysregulation-an overview. Semin Immunopathol. (2018) 40:49–64. doi: 10.1007/s00281-017-0663-8 29327071 PMC5794843

[62] Domínguez Del Olmo P Herraiz I Villalaín C Galindo A Ayala JL . Predictive modeling of complications arising from early-onset preeclampsia in pregnant women. Womens Health (Lond). (2025) 21:17455057251348978. doi: 10.1177/17455057251348978 40686311 PMC12280542

[63] Pan X Peng J Chen Y Di X Li P Zhang G . sFlt-1/PlGF ratio thresholds for diagnosing pre-eclampsia in pregnant women with high blood pressure. Ultrasound Obstet Gynecol. (2025) 66:631–40. doi: 10.1002/uog.70075 40992360 PMC12579772

[64] Qi L Teschendorff AE . Cell-type heterogeneity: Why we should adjust for it in epigenome and biomarker studies. Clin Epigenet. (2022) 14:31. doi: 10.1186/s13148-022-01253-3 35227298 PMC8887190

[65] Parapob N Luewan S Kamlungkuea T Tongsong T . Oxidative stress in pathogenesis of preeclampsia: mechanistic and clinical insights. Antioxidants (Basel). (2026) 15:387. doi: 10.3390/antiox15030387 41897533 PMC13023569

[66] Musalem P Bascur N Sepúlveda RA Krall P Lazcano A Navarro G . Diagnosis and treatment of complement-mediated thrombotic microangiopathies: consensus of the Genetic Diseases Committee of the Chilean Society of Nephrology. BMC Nephrol. (2026) 27:21. doi: 10.1186/s12882-025-04739-3 41495696 PMC12784606

[67] Nirupama R Divyashree S Janhavi P Muthukumar SP Ravindra PV . Preeclampsia: pathophysiology and management. J Gynecol Obstet Hum Reprod. (2021) 50:101975. doi: 10.1016/j.jogoh.2020.101975 33171282

[68] Story CM Chaturvedi S Gerber GF . Hematologic disorders in pregnancy: the role of the complement system. Expert Rev Hematol. (2026) 19:491–506. doi: 10.1080/17474086.2026.2634997 41784357 PMC13370807

[69] Fakhouri F Scully M Ardissino G Al-Dakkak I Miller B Rondeau E . Pregnancy-triggered atypical hemolytic uremic syndrome (aHUS): a global aHUS registry analysis. J Nephrol. (2021) 34:1581–90. doi: 10.1007/s40620-021-01025-x 33826112 PMC8494679

[70] Smith-Jackson K Harrison RA . Alternative pathway activation in pregnancy, a measured amount "complements" a successful pregnancy, too much results in adverse events. Immunol Rev. (2023) 313:298–319. doi: 10.1111/imr.13169 36377667 PMC10100418

[71] Sperati CJ . How I treat complement-mediated TMA. Clin J Am Soc Nephrol. (2022) 17:452–4. doi: 10.2215/cjn.13581021 35074846 PMC8975032

[72] Halacova N Brndiarova M Slenker B Ruzinak Bobcakova A Schniederova M Markocsy A . Complement inhibitors and the risk of (breakthrough) infections—critical analysis and preventive strategies. Biologics. (2026) 6:3. doi: 10.3390/biologics6010003 30654563

[73] Baroutis D Koukoumpanis K Tzanis AA Theodora M Rizogiannis K Bairaktaris D . Low-molecular-weight heparin in preeclampsia: effects on biomarkers and prevention: a narrative review. Biomedicines. (2025) 13:2337. doi: 10.3390/biomedicines13102337 41153624 PMC12561593

[74] Tong S Kaitu'u-Lino TJ Hastie R Brownfoot F Cluver C Hannan N . Pravastatin, proton-pump inhibitors, metformin, micronutrients, and biologics: new horizons for the prevention or treatment of preeclampsia. Am J Obstet Gynecol. (2022) 226:S1157–70. doi: 10.1016/j.ajog.2020.09.014 32946849

[75] Gera P Frishman WH Aronow WS . The use of statins during pregnancy in patients diagnosed with preeclampsia: a systematic review. Cardiol Rev. (2025). doi: 10.1097/crd.0000000000000906 40126023

[76] Kolev M Barbour T Baver S Francois C Deschatelets P . With complements: C3 inhibition in the clinic. Immunol Rev. (2023) 313:358–75. doi: 10.1111/imr.13138 36161656

[77] Iannaccone A Reisch B Mavarani L Darkwah Oppong M Kimmig R Mach P . Soluble endoglin versus sFlt-1/PlGF ratio: detection of preeclampsia, HELLP syndrome, and FGR in a high-risk cohort. Hypertens Pregnancy. (2022) 41:159–72. doi: 10.1080/10641955.2022.2066119 35475405

[78] Hamad OA Ekdahl KN Nilsson PH Andersson J Magotti P Lambris JD . Complement activation triggered by chondroitin sulfate released by thrombin receptor-activated platelets. J Thromb Haemost. (2008) 6:1413–21. doi: 10.1111/j.1538-7836.2008.03034.x 18503629 PMC2673520

[79] Desikan SP Sperati CJ . Do we still need plasma exchange to treat TMA? Yes. Nephrol Dial Transplant. (2025) 40:2224–6. doi: 10.1093/ndt/gfaf149 40795238

[80] Demel I Honová S Vaňková K Gumulec J Šimetka O . Overview of pregnancy-associated thrombotic microangiopathies–a practical guidance. Bratislava Med J. (2025) 126, 3691–3708. doi: 10.1007/s44411-025-00361-9 30311153

[81] Azoulay E Zuber J Bousfiha AA Long Y Tan Y Luo S . Complement system activation: bridging physiology, pathophysiology, and therapy. Intensive Care Med. (2024) 50:1791–803. doi: 10.1007/s00134-024-07611-4 39254734

[82] Özcan M Peker S . Preeclampsia prediction via machine learning: a systematic literature review. Health Syst (Basingstoke). (2025) 14:208–22. doi: 10.1080/20476965.2024.2435845 40837108 PMC12364110

[83] Bachnas MA Andonotopo W Prabowo W Yuliantara EE Pramono MBA Dewantiningrum J . Precision medicine in perinatal cardiorespiratory disease: a systematic review of multi-omics, genetic profiling, and pharmacogenomics for maternal–fetal risk assessment. Sarvodaya Int J Med. (2026) 2:15–29. doi: 10.4103/sijm.sijm_50_25 42081533

